# Hospital Officers' Association

**Published:** 1924-04

**Authors:** 


### ASSOCIATION OF HOSPITAL OFFICERS.

The Incorporated Association of Hospital Officers, which was founded in 1902, and incorporated in 1910, announces that its first annual conference will take place on May 22, 23 and 24, at the Central Hall, Westminster. The first two days will be devoted to the business of the conference, and the third day to sightseeing, visits to hospitals, etc. Mr. H. J. Waring, F.R.C.S., Vice-Chancellor of the University of London (Senior Surgeon, St. Bartholomew's Hospital), has consented to read a paper on "Voluntary Hospitals and Paying Patients." Three other papers will be read—one by a member of each Provincial Branch, and one by a London member. The Annual Dinner of the Association will be held at the Holborn Restaurant on Thursday, May 22, when Viscount Cave will be the guest of the evening. Arrangements have been made for the issue of reduced fare tickets to provincial members and friends at the rate of a fare and a third for the return journey. Hotel accommodation has been reserved and private hospitality will be offered to those who do not desire to stay in hotels. The President of the Association, Mr. H. L. Eason, C.B., C.M.G., M.D., M.S., will be President of the conference, and will take the chair at the Annual Dinner. The Hon. Secretary of the Association (Mr. J. E. Stone) will supply full information on application to 28, Bedford Square, W.C. 1.

